# A case of recurrent massive thickening of the gastric wall caused by pancreatitis of the gastric ectopic pancreas: Detailed pathogenesis based on imaging

**DOI:** 10.1002/deo2.188

**Published:** 2022-11-24

**Authors:** Shun Sasaki, Kazuhiro Ota, Ryo Tanaka, Yoshiro Imai, Yuichi Kojima, Hiroshi Akutagawa, Taro Iwatsubo, Noriaki Sugawara, Akitoshi Hakoda, Hironori Tanaka, Yosuke Mori, Sang‐Woong Lee, Toshihisa Takeuchi, Hiroki Nishikawa

**Affiliations:** ^1^ Second Department of Internal Medicine Osaka Medical and Pharmaceutical University Osaka Japan; ^2^ Endoscopy Center Osaka Medical and Pharmaceutical University Hospital Osaka Japan; ^3^ Department of General and Gastroenterological Surgery Osaka Medical and Pharmaceutical University Osaka Japan; ^4^ Department of Pathology Osaka Medical and Pharmaceutical University Osaka Japan

**Keywords:** computed tomography, ectopic pancreas, gastroscopy, histopathology, pancreatitis

## Abstract

A 40‐year‐old Japanese male presented with epigastric pain and loss of appetite at a general hospital three years ago. Computed tomography revealed massive thickening of the gastric wall, and gastroscopy revealed diffuse erythema and edematous thickening of the gastric mucosa. Thereafter, epigastric pain and gastric wall thickening recurred frequently, causing an inability to intake food. Conservative treatment was marginally effective; therefore, a distal gastrectomy was performed. Postoperatively, the patient resumed food intake without complications. Histopathological examination of the surgical specimen revealed Heinrich type 1 gastric ectopic pancreas (EP) with pancreatitis. In this case, the gastric wall's massive thickening was caused by gastric EP's pancreatitis. Although there are some reports of pancreatitis of gastric EP, there are no detailed reports of endoscopic findings, including endoscopic ultrasonography and the disease progression. Recurrent pancreatitis of EP leads to forming a septum within the gastric wall, resulting in a hematoma. Eventually, irreversible narrowing of the gastric lumen may occur, as observed in the present case. We consider this an important case report presenting detailed pathogenesis supported by endoscopic and pathohistological findings of surgical specimens. Our study will help in the early diagnosis and better management of the condition.

## INTRODUCTION

An ectopic pancreas (EP) is defined as pancreatic tissue that lacks anatomical continuity with the normal pancreas and has different hemodynamics from the pancreas proper. EP occurs in 0.5%–13% of autopsy cases. Its prevalence is 27.7% in the duodenum, 25.5% in the stomach, and 15.0% in the jejunum, and it also occurs in the upper gastrointestinal tract.^1^ Although most EPs remain asymptomatic, any disease inflicting the pancreas proper can also occur in the EP. The prevalence and pathogenesis of EP presenting with clinical symptoms are unclear, even in the latest papers, because there are few published reports on the asymptomatic EP.^2^

It was reported that the clinical symptoms of the pancreatitis of gastric EP are non‐specific and include abdominal pain, nausea, vomiting, anorexia, and weight loss.^3^, ^4^ Specific endoscopic findings of gastric EP are not well characterized; hence, diagnosing gastric EP using gastroscopy alone is difficult.

Herein, we report a case of pancreatitis of gastric EP with gradual exacerbation. We consider this an important case report presenting pathogenesis supported by endoscopic and pathohistological findings of surgical specimens.

## CASE PRESENTATION

**FIGURE 1:**
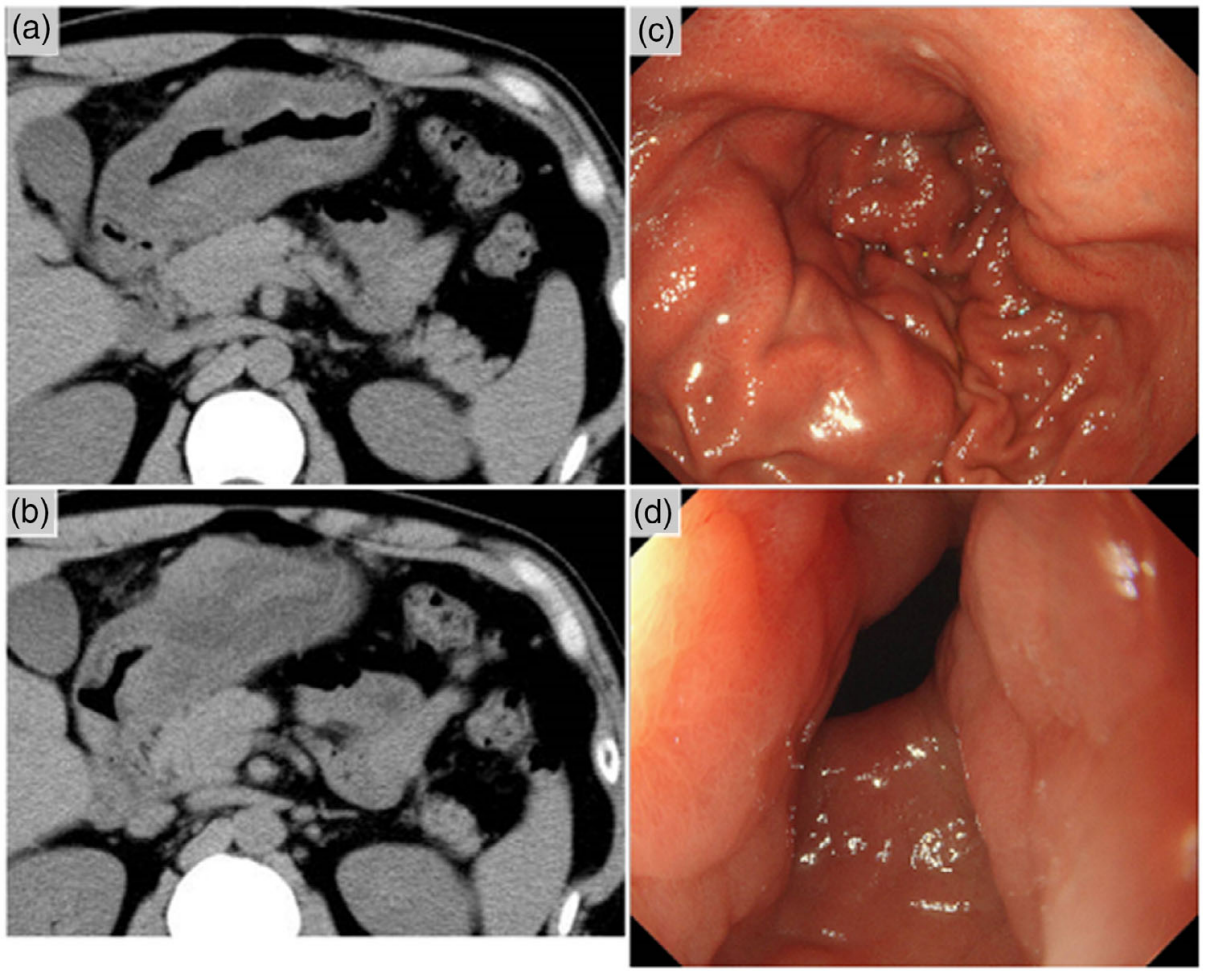
Imaging findings at the initial onset of the pancreatitis of gastric ectopic pancreas. Computed tomography revealed massive thickening of the gastric wall (a,b), and gastroscopy revealed diffuse erythema and edematous thickening of the gastric mucosa (c,d).

**FIGURE 2:**
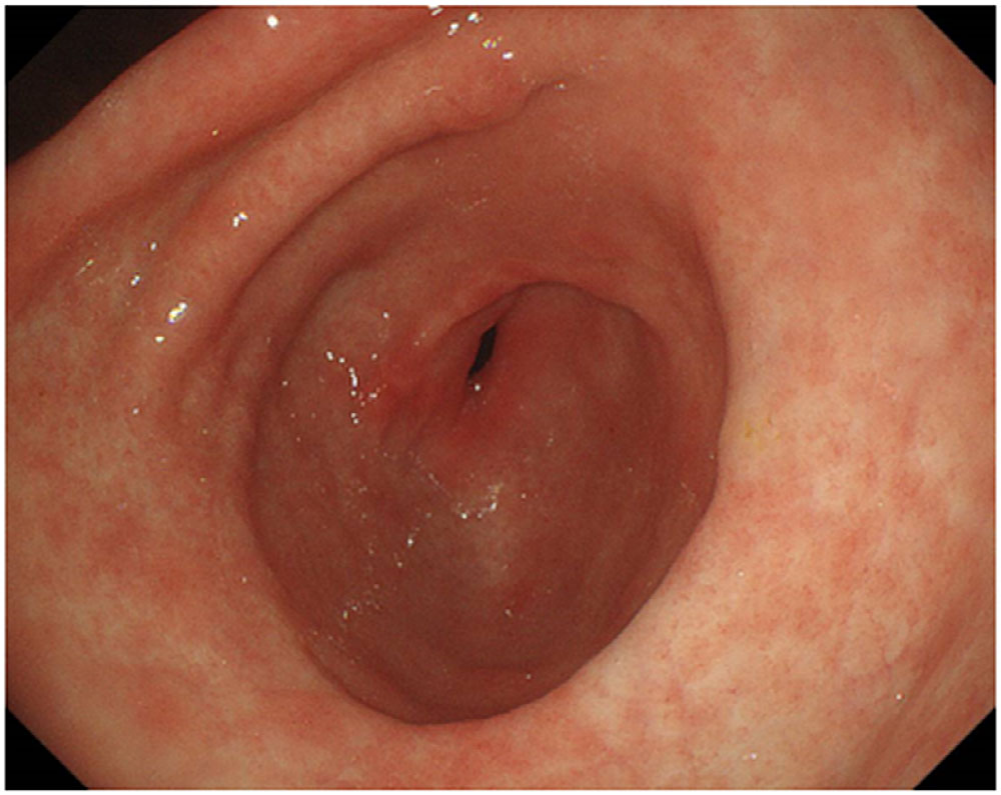
The gastroscopic findings 5 months from the initial onset when the patient was asymptomatic. The gastric wall thickening, as per gastroscopy, was absent.

A 40‐year‐old Japanese male with a history of atopic dermatitis and bronchial asthma presented with epigastric pain and loss of appetite at a general hospital three years ago. He had drunk between 700 and 1000 ml of beer daily, five days a week. Blood tests at the initial visit showed that triglycerides were within the reference range; triglycerides 62 mg / dL (Normal range, 55–150 mg/dL). Computed tomography (CT) revealed massive gastric wall thickening (Figure 1a,b). Gastroscopy revealed diffuse erythema and edematous thickening of the gastric mucosa, mainly in the distal side of the stomach (Figure 1c,d). Although scirrhous gastric cancer was suspected, the endoscopic biopsy did not prove the presence of carcinoma cells. One month later, he was referred to our institution for further examination and a definite diagnosis. During our examination, the epigastric pain had alleviated, and gastroscopy revealed that the swelling of the gastric mucosa had subsided. There were no endoscopic findings suggestive of gastric cancer (Figure S1). Chronic gastritis associated with *Helicobacter pylori* was noted and was successfully eradicated. Thereafter, the patient remained asymptomatic for one year. Figure 2 showed the gastroscopic findings 5 months after onset when he was asymptomatic, and the gastroscopic findings of gastric wall thickening were absent.

One year after the primary onset, the epigastric symptoms and gastric wall thickening recurred, as revealed by CT and endoscopic findings (Figure S2). The symptoms were refractory to 20 mg of vonoprazan, a potassium‐competitive acid blocker. The cause of the symptoms, which persisted for about 5 months, remained unknown, following which the symptoms subsided. The third onset occurred exactly two years after the initial onset. Symptoms appeared every year, and we suspected seasonal allergy as the etiology because he was complicated with bronchial asthma and atopic dermatitis. Endoscopic biopsy showed no eosinophilic infiltration of the gastric mucosa, but the patient was administered steroids and antihistamines as a treatment for eosinophilic gastritis; however, these medications were refractory.

**FIGURE 3:**
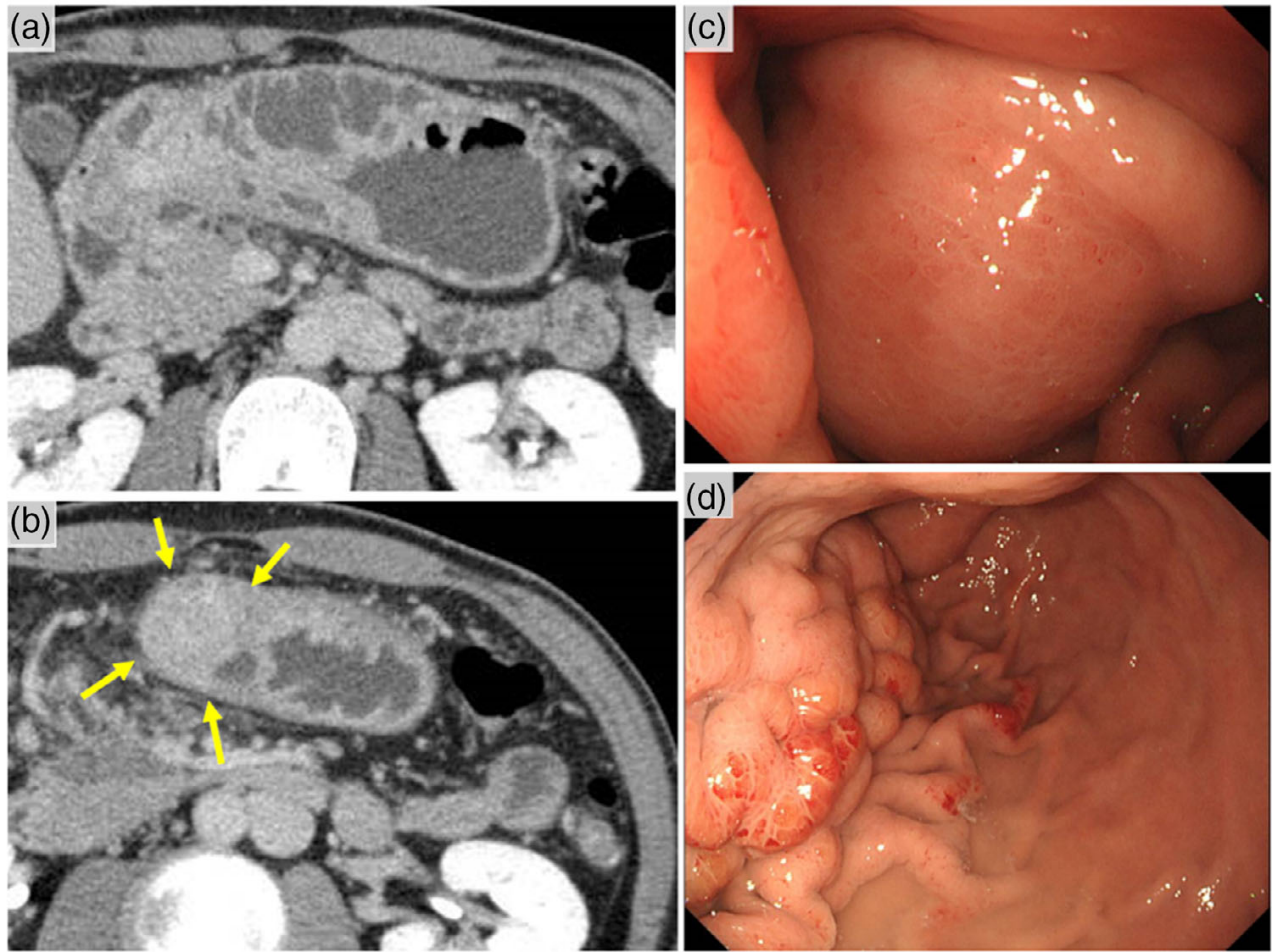
Twenty‐three months after the initial onset, he was hospitalized due to severe vomiting, preventing food intake. Contrast‐enhanced abdominal computed tomography revealed a massive gastric wall thickening, and gastric intramural lesion (arrows) (a,b) and gastroscopy revealed edematous gastric mucosa with edematous stenosis (c,d).

**FIGURE 4:**
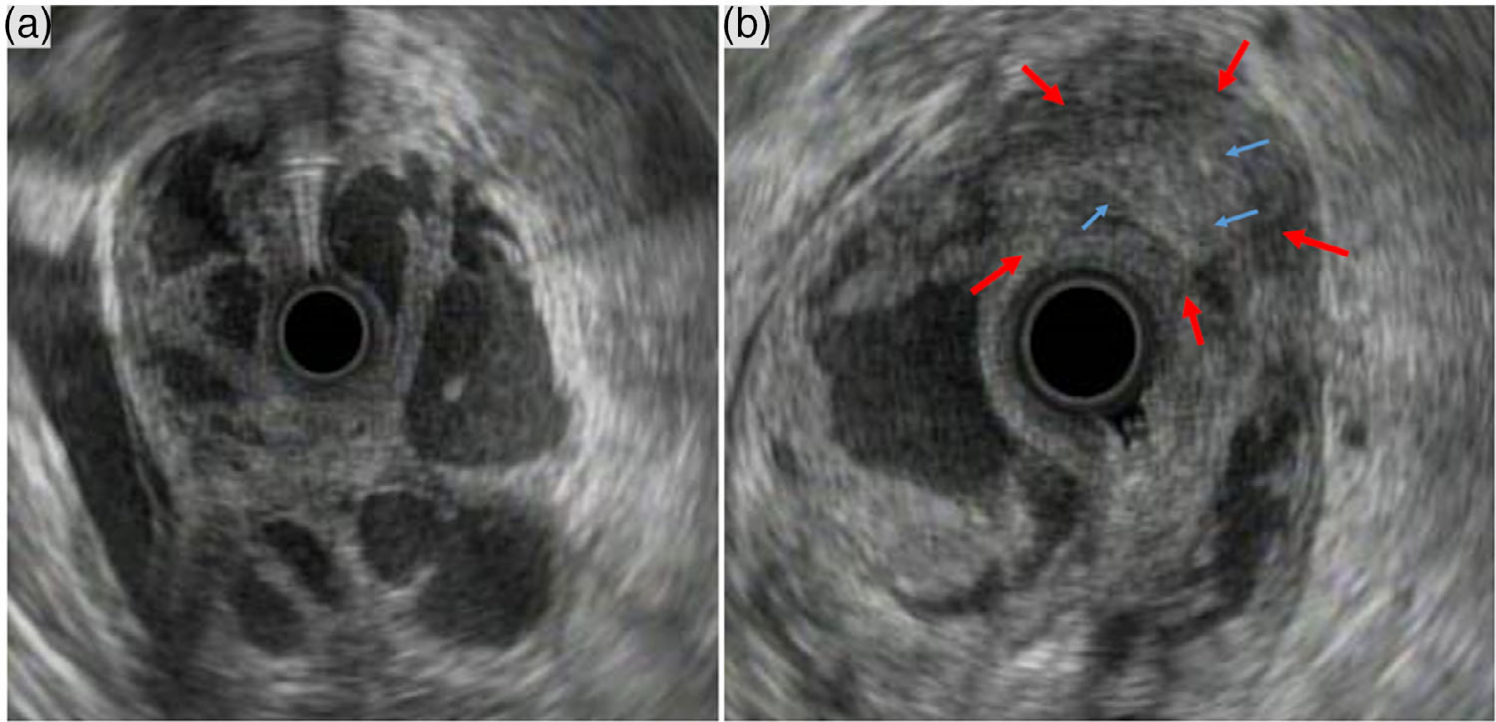
Endoscopic ultrasonography findings. Multiple submucosal cysts at the gastric body and antrum (a). At the gastric antrum, the submucosal tumor within the muscularis propria (b, red arrows) with scattered linear echoes inside (b, blue arrows) were the findings consistent with the ectopic pancreas.

Six months later, he was hospitalized due to severe vomiting, preventing food intake. Contrast‐enhanced abdominal CT revealed massive gastric wall thickening and a gastric intramural mass; contrast‐enhanced CT was not performed until this time due to the history of bronchial asthma (Figure 3a,b). Gastroscopy revealed edematous gastric mucosa with edematous stenosis (Figure 3c,d). In particular, the gastric antrum was severely affected compared to the gastric body. Scirrhous gastric cancer was suspected; however, an endoscopic mucosal biopsy did not confirm the diagnosis. Similarly, laparoscopy revealed no obvious evidence of gastric cancer, and the greater omentum firmly adhered to the gastric wall (Figure S3). Endoscopic ultrasonography (EUS) revealed a submucosal tumor within the muscularis propria of the gastric antrum, with scattered linear echoes. These findings were with typical EP findings. (Figure 4). We suspected pancreatitis of EP of the gastric wall. The presence of pancreatic hyperamylasemia and hyperlipasemia also supported this diagnosis (Table S1). Since conservative treatment was limited, a distal gastrectomy was performed. The primary site of the pathology was thought to be on the distal side of the stomach because there were diffuse erythema and edematous thickening of the gastric mucosa, mainly in the distal side of the stomach in gastroscopy as well as EUS findings. Although there was certainly a little possibility of developing pancreatitis of EP in the residual stomach, distal gastrectomy was selected because the main site of mucosal thickening was distal to the stomach based on endoscopic and EUS findings. Postoperatively, the serum amylase levels normalized, and food intake could be resumed (Figure S4). Histopathological examination of the surgical specimen revealed gastric EP (Heinrich type 1) with pancreatitis (Figure S5).

## DISCUSSION

In this rare case, the gastric wall's massive thickening was caused by gastric EP's pancreatitis. Although there are some reports of pancreatitis of gastric EP, there are no detailed reports of endoscopic findings, including endoscopic ultrasonography and the disease progression.

The diagnosis was challenging since we had not encountered any cases of gastric EP. A preoperative diagnosis of gastric wall thickening associated with EP was made, and the surgery was performed. The decisive factors for the preoperative diagnosis of the pancreatitis of EP were: the presence of a mass in the gastric wall as revealed by contrast‐enhanced CT, hyperamylasemia, and the findings on ultrasound endoscopy.

Specific endoscopic findings of the pancreatitis of gastric EP have not been reported. In the first endoscopy in the present case, there was no specific abnormality on the mucosal surface, barring redness and swelling of the mucous membranes and narrowing of the gastric antral space due to the thickening of the submucosa. As days progressed, wall thickening in the gastric antecubital area decreased, and there were no abnormal findings. Similar gastrointestinal endoscopic findings recurred at the onset of pancreatitis in the gastric wall.

During the progression of the pancreatitis of EP, we considered that the clinical course of CT findings was specific. The thickening of the gastric wall at the time of initial onset was uniform. However, a cyst‐like low‐density area within the gastric wall was observed after the second examination. Contrast‐enhanced CT clearly showed a blood‐rich mass within the gastric wall. These CT findings may be specific to the pancreatitis of EP. Endoscopic ultrasonographic findings also revealed cyst‐like hypoechoic and submucosal mass consistent with EP. Repeated pancreatitis within the gastric wall probably caused the formation of a septum within the gastric wall. By contrast with the resected specimen, it was clear that the cyst‐like lesion was a hematoma.

Serum amylase levels were slightly elevated but were measured only during onset periods. The apparent postoperative decrease suggested that the patient had hyperamylasemia, reflecting pancreatitis of gastric EP. To improve the accuracy of preoperative diagnosis, frequent screening, including non‐onset periods, in addition to initial measurement during onset, should be considered.

The pancreatitis of gastric EP is a benign disease, and surgery is not essential in the absence of severe clinical symptoms, as in the present case. However, even if surgery is not performed, a thorough examination should be performed, and the possibility of ectopic pancreatic cancer should be ruled out.^5^ In this case, the acute pancreatitis of the EP may have resulted from obstruction of the pancreatic duct in the EP for some reason, but the exact cause is unknown. Alcohol ingestion may have been involved.

In conclusion, recurrent pancreatitis of EP leads to forming of a septum within the gastric wall, resulting in a hematoma. Eventually, irreversible narrowing of the gastric lumen may occur, as observed in the present case. We consider this an important case report presenting detailed pathogenesis supported by endoscopic and pathohistological findings of surgical specimens. Our study will help in the early diagnosis and better management of the condition.

## CONFLICT OF INTEREST

The authors declare that they have no conflict of interest.

## Supporting information


**Figure S1** Endoscopic imaging of pancreatitis gastric EP at 1 month later from the initial onset

**Figure S2** Computed tomographic and endoscopic findings when reoccurred epigastric symptoms and gastric wall thickening at 1 year after primary onset

**Figure S3** Laparoscopic findings

**Figure S4** Serum amylase levels at several attacks

**Figure S5** Surgical specimen after formalin fixation of distal gastrectomy and its Hematoxylin‐eosin‐stained microscopic images

**Table S1** Blood test results
