# Supplementary figures and images for: A case of recurrent massive thickening of the gastric wall caused by pancreatitis of the gastric ectopic pancreas: Detailed pathogenesis based on imaging
Source: DEN Open. 2022 Nov 24;3(1):e188. doi: 10.1002/deo2.188 (PMC9691903; doi:10.1002/deo2.188)

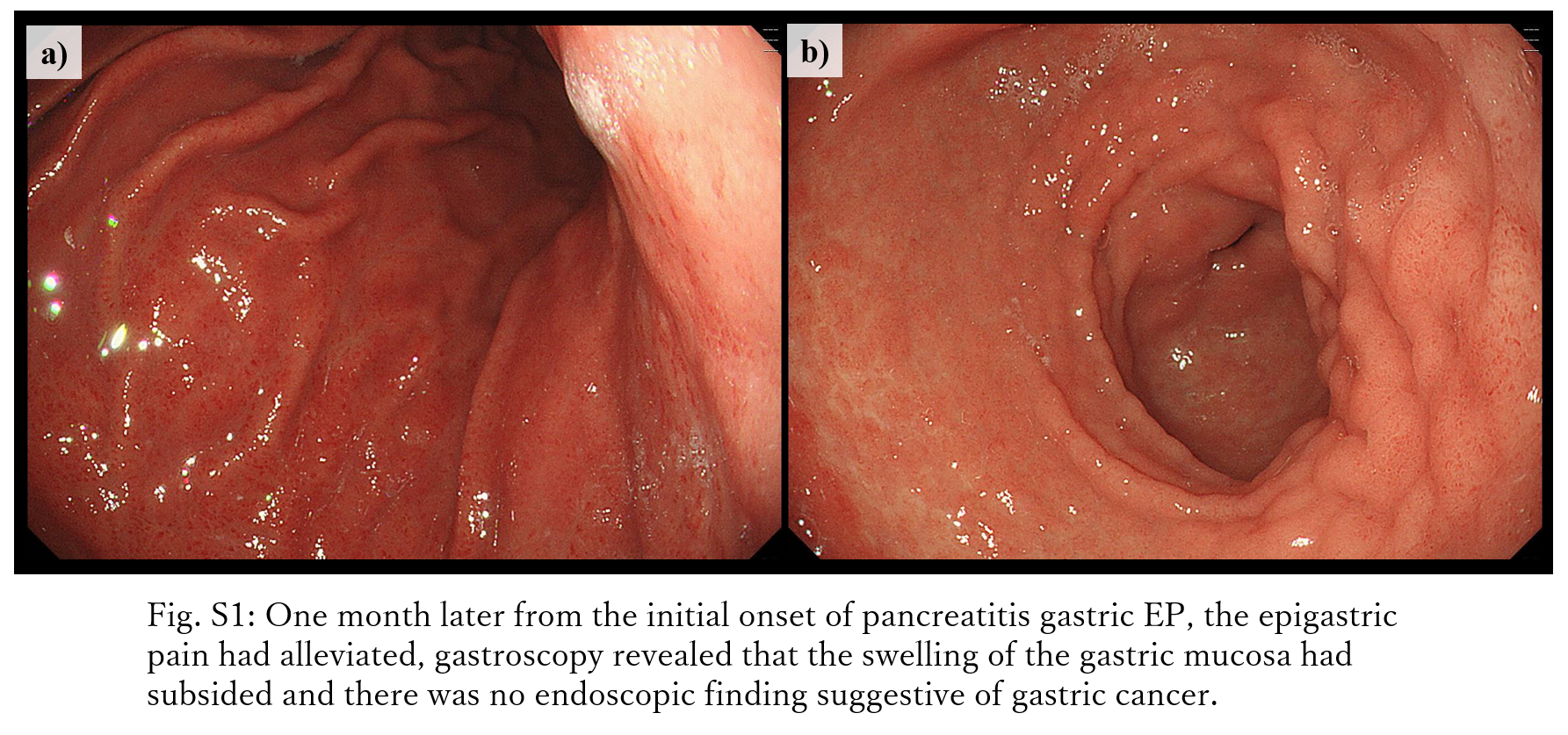

Supplement: Supplementary file 1 — Figure S1 Endoscopic imaging of pancreatitis gastric EP at 1 month later from the initial onset [file DEO2-3-e188-s005.tif]

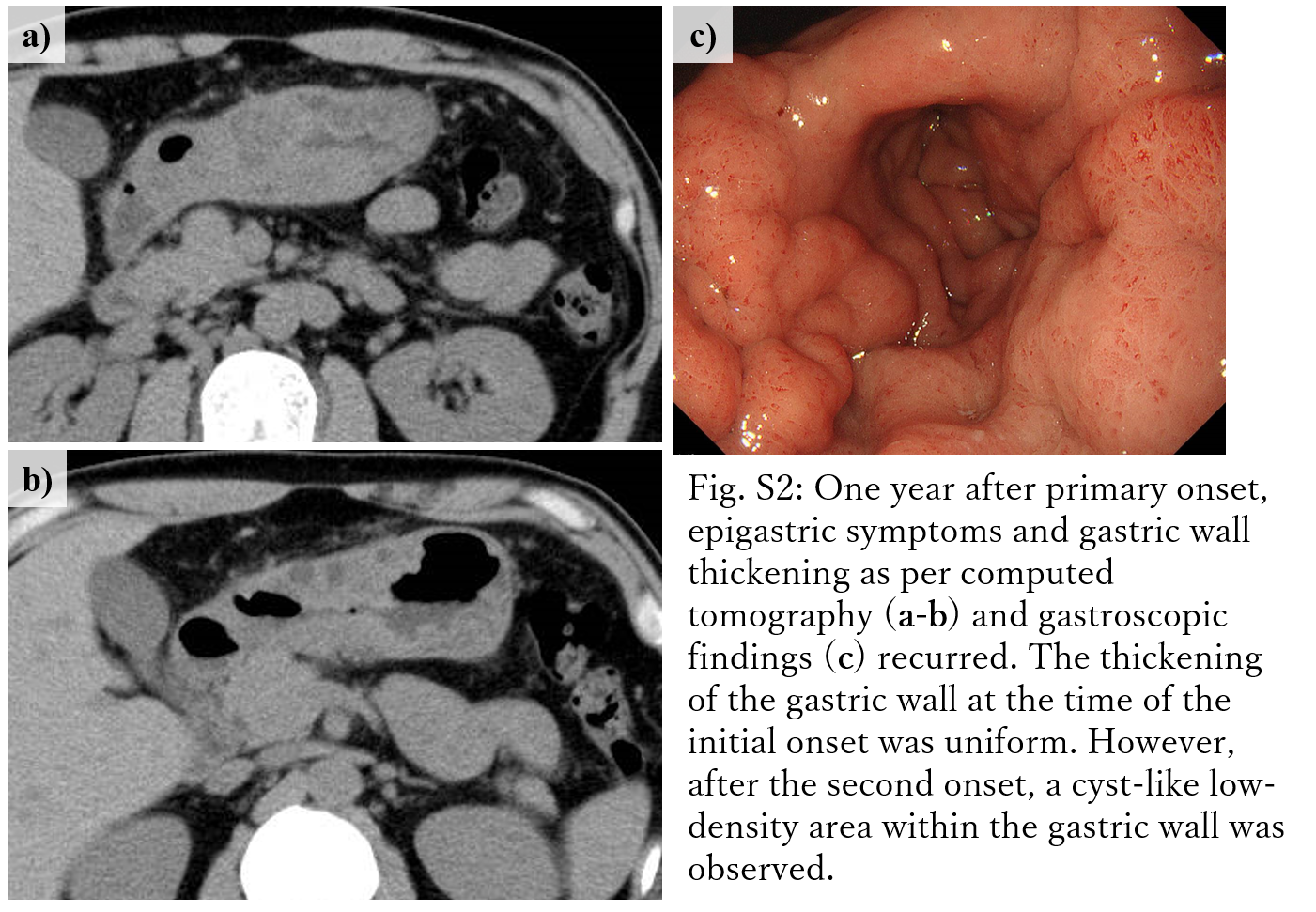

Supplement: Supplementary file 2 — Figure S2 Computed tomographic and endoscopic findings when reoccurred epigastric symptoms and gastric wall thickening at 1 year after primary onset [file DEO2-3-e188-s004.tif]

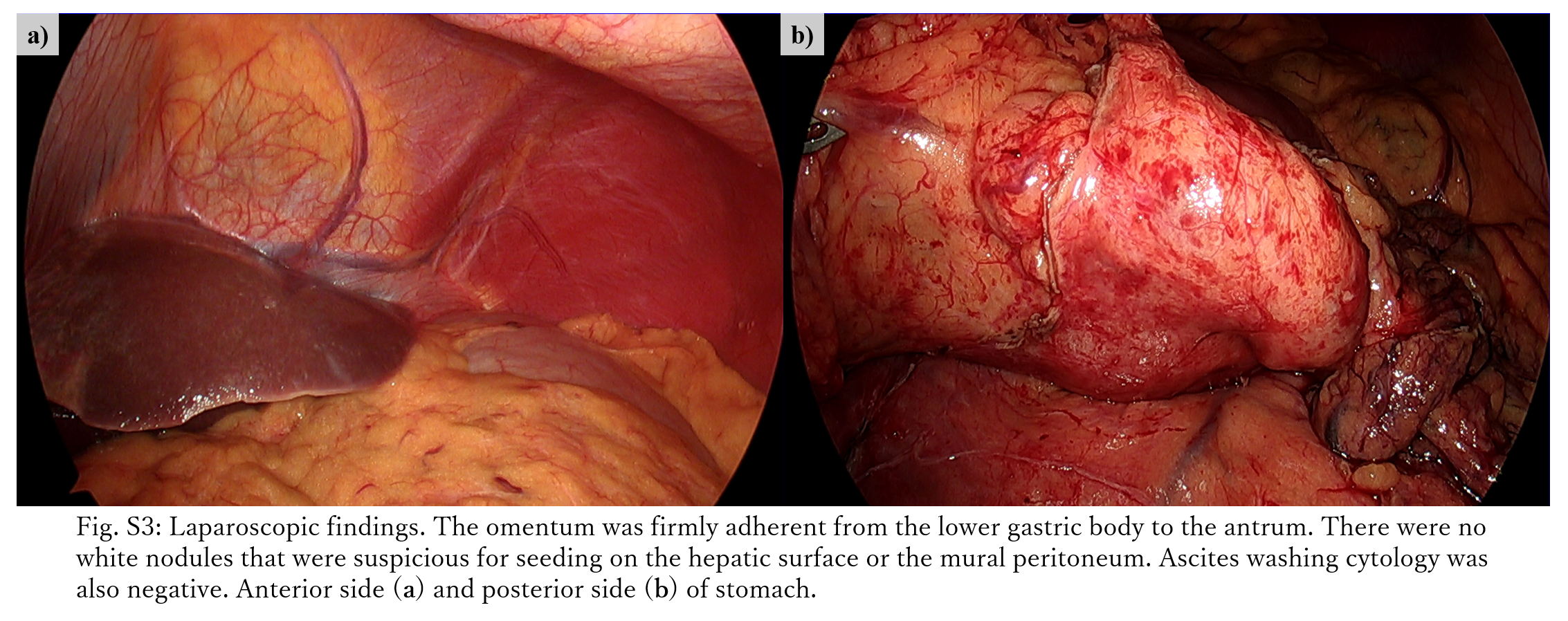

Supplement: Supplementary file 3 — Figure S3 Laparoscopic findings [file DEO2-3-e188-s001.tif]

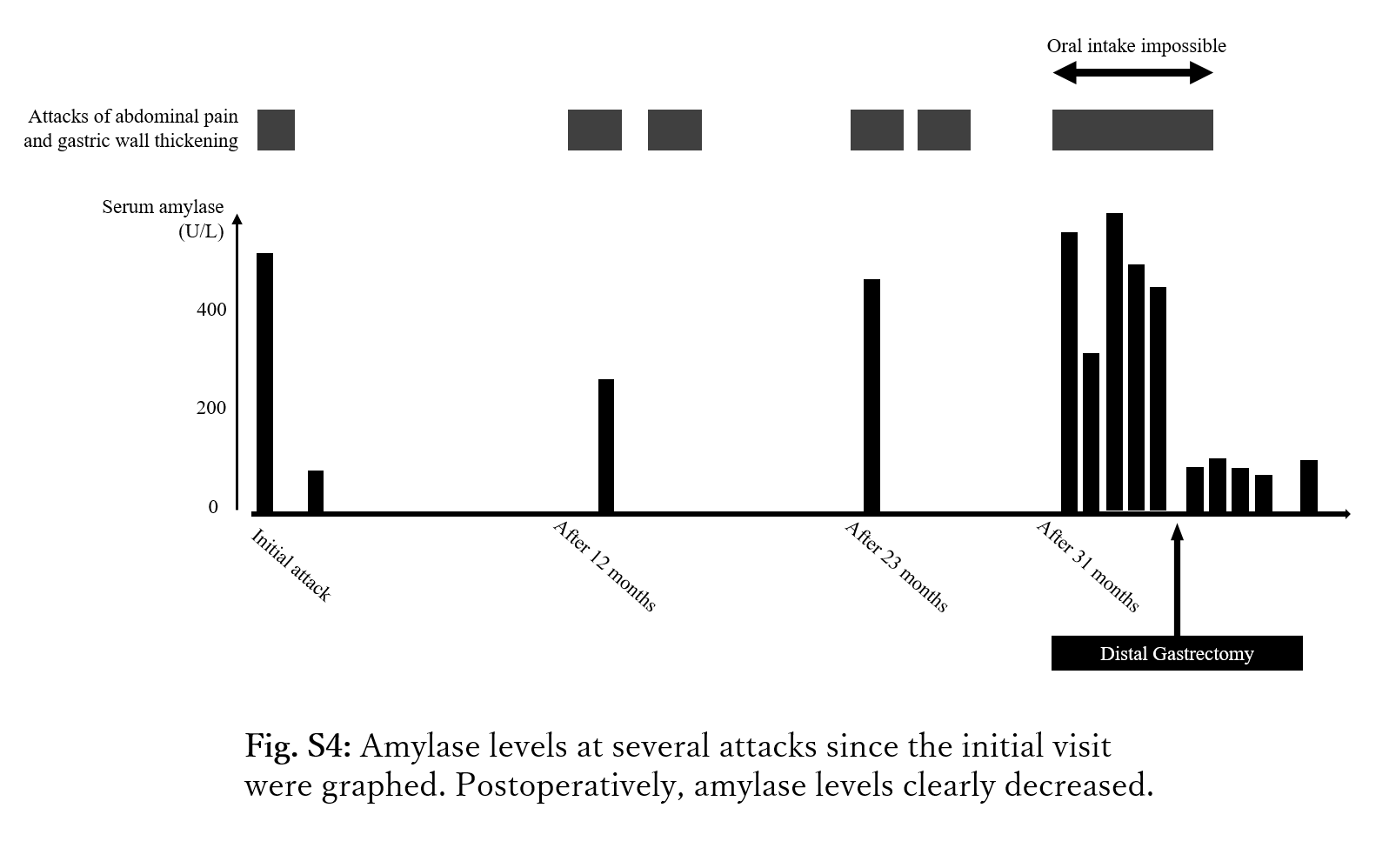

Supplement: Supplementary file 4 — Figure S4 Serum amylase levels at several attacks [file DEO2-3-e188-s003.tif]

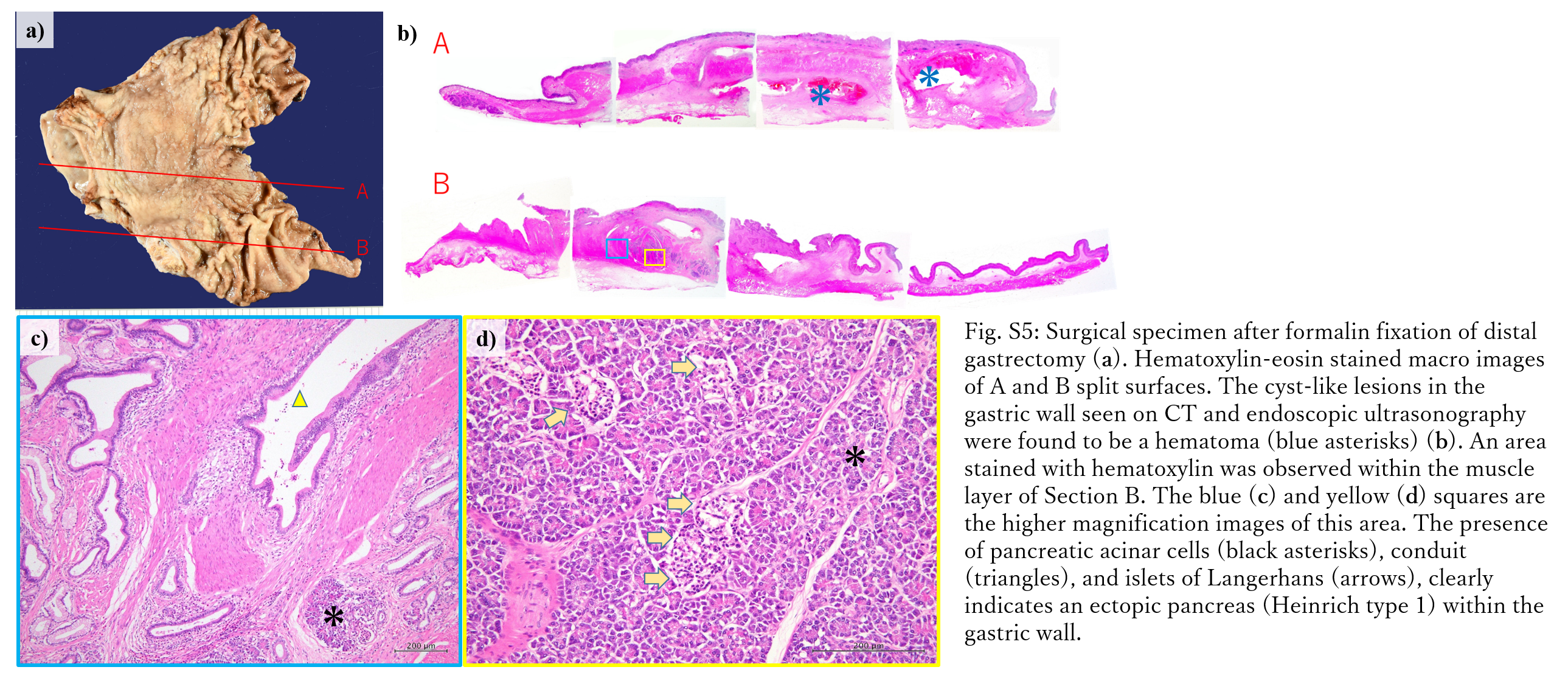

Supplement: Supplementary file 5 — Figure S5 Surgical specimen after formalin fixation of distal gastrectomy and its Hematoxylin‐eosin‐stained microscopic images [file DEO2-3-e188-s002.tif]
