# Supplementary material for: A case of recurrent massive thickening of the gastric wall caused by pancreatitis of the gastric ectopic pancreas: Detailed pathogenesis based on imaging
Source: DEN Open. 2022 Nov 24;3(1):e188. doi: 10.1002/deo2.188 (PMC9691903; doi:10.1002/deo2.188)
Supplement: Supplementary file 6 — Table S1 Blood test results [file DEO2-3-e188-s006.docx]

Table S1. Blood test results.

| Items (unit) | Measured value | Normal range from the Osaka Medical and Pharmaceutical University Hospital |
| --- | --- | --- |
| White blood cell counts (/µL) | 6,230 | 3300–8600 |
| Red blood cell counts (×10^4^/µL) | 428 | 435–555 |
| Hemoglobin (g/dL) | 12.3 | 13.7–16.8 |
| Platelet count (×10^3^/µL) | 271 | 158–348 |
| Total protein (g/L) | 6.5* | 6.6–8.1 |
| Albumin (g/L) | 3.8* | 4.1–5.1 |
| Aspartate aminotransferase (U/L) | 12 | 13–30 |
| Alanine aminotransferase (U/L) | 10 | 10–42 |
| Lactate dehydrogenase (U/L) | 226* | 124–222 |
| Alkaline phosphatase (U/L) | 69 | 38–113 |
| Amylase (U/L) | 547* | 44–132 |
| ** Amylase (U/L) | 314* | 44-132 |
| ** Pancreatic amylase (U/L) | 279* | 14-41 |
| ** Lipase (U/L37℃) | 238* | 13-49 |
| γ-glutamyl transpeptidase (U/L) | 7 | 13–64 |
| Blood urea nitrogen (mg/dL) | 5 | 8–20 |
| Creatinine (mg/dL) | 0.62 | 0.65–1.07 |
| Carcinoembryonic antigen (ng/mL) | 0.7 | <0.5 |
| Carbohydrate antigen 19-9 (ng/mL) | 14.7 | <37.0 |

* outside the reference range. ** different samples collected 2 weeks later.
